# Network-based multi-omics approaches to identify molecular signatures associated with pregnancy status in beef heifers

**DOI:** 10.3389/fgene.2026.1794156

**Published:** 2026-04-20

**Authors:** T. Cody Brown, Priyanka Banerjee, Paul W. Dyce, Shollie Falkenberg, Soren P. Rodning, Wellison J. S. Diniz

**Affiliations:** 1 Department of Animal Sciences, Auburn University, Auburn, AL, United States; 2 Department of Pathobiology, College of Veterinary Medicine, Auburn University, Auburn, AL, United States

**Keywords:** beef heifers, fertility, multi-omics, networks, proteomics, transcriptomics

## Abstract

Fertility is a multifactorial trait and a key determinant of productivity and sustainability in beef cattle production. Identifying molecular mechanisms and biomarkers associated with fertility could improve the prediction of reproductive potential in beef heifers. Herein, by combining transcriptomic and proteomic data from peripheral white blood cells (PWBCs) collected before the time of artificial insemination (AI), we investigated molecular differences between fertile and subfertile beef heifers (n = 6 per group) classified based on their reproductive outcomes. RNA-Sequencing and untargeted proteomics identified 230 differentially expressed genes (DEGs; *P* ≤ 0.05 and |log2FC| ≥ 0.5) and 70 differentially abundant proteins (DAPs; *P* ≤ 0.05) between groups. Over-representation analyses revealed that these molecules were associated with cell cycle regulation, metabolism, and immune-related pathways, including chemokine and JAK-STAT signaling (*P* ≤ 0.01). Data integration revealed limited overlap between DEGs and DAPs (*UROS, KIFC3, DHRSX,* and *NPL*). Among these, *NPL* expression was previously reported to be progesterone-responsive, supporting its potential role in early pregnancy establishment. Network analyses revealed distinct regulatory patterns between groups (|r ≥ 0.95| and *P* ≤ 0.05). At the transcript level, subfertile heifers exhibited increased connectivity, indicating potential compensatory transcriptional rewiring. We identified 92 regulatory impact factor (RIF) genes with potential modulatory roles, including *ESR1*. Epigenetic transcription factors, including *MBD1, MBD2,* and *SMARCE1,* were also rewired, suggesting an interplay between hormone signaling and chromatin regulation that modulates transcript expression and consequently fertility outcomes. Our results show that PWBCs reflect systemic molecular changes associated with fertility status and represent a promising, non-invasive source for biomarker discovery. This integrative multi-omics approach provided novel insights into the regulatory networks underlying fertility in beef heifers, highlighting the value of integrating multi-omics to identify key pathways and molecular targets to improve reproductive efficiency in beef production systems.

## 1 Introduction

A key priority in the beef industry is to improve the early prediction of fertility potential, thereby reducing the economic losses associated with pregnancy failure (Ealy, 2020). Despite high fertilization rates being reported in beef cattle, pregnancy rates are only approximately 50% 1 month after a single insemination (Reese et al., 2020). Combining artificial insemination (AI) with natural breeding (NB), pregnancy rates can achieve up to 85% or more in beef heifers (Moorey and Biase, 2020); however, a portion of the herd still fails to conceive by the end of the breeding season. Non-pregnant heifers after a breeding season represent a significant loss of the producer’s investment of time and resources, reducing the overall efficiency of the production system (Hindman and Engelken, 2021; Kertz et al., 2023). Traditionally, producers use traits such as age, body condition (BCS), and reproductive tract scores (RTS) to evaluate reproductive maturity and select replacement heifers (Holm et al., 2009; Markusfeld et al., 1997). While there are benefits to using such approaches (Gutierrez et al., 2014), their ability to discriminate or predict heifers’ reproductive outcomes remains limited, as a portion of animals still fail to conceive despite selection (Dickinson et al., 2019).

As heifers develop, they undergo morphological and hormonal changes that culminate in the onset of puberty (Kelly et al., 2022). This process is regulated by a complex, coordinated network of genes, proteins, and hormones that prepares the reproductive system for establishing pregnancy (Perry, 2012; Poliakiwski et al., 2025). Similarly, successful conception and establishment of pregnancy depend on finely tuned maternal adjustments that ensure proper communication between the embryo and the reproductive tract (Kertz et al., 2023; Pohler et al., 2020; Poliakiwski et al., 2025). These adjustments involve hormonal regulation, immune modulation, and changes in uterine receptivity, as well as molecular and cellular remodeling within the endometrium, all of which create a receptive environment to support embryo recognition, implantation, and development (Raliou et al., 2025; Rocha et al., 2025; Silva et al., 2023). A growing number of studies have examined the endometrium to understand better the hormonal and molecular changes underlying the establishment of pregnancy and uterine receptivity (Binelli et al., 2015; Killeen et al., 2014; Minten et al., 2013). These studies have contributed significantly to our understanding; however, endometrial sampling remains invasive and impractical for routine application in beef production systems.

Physiological and molecular changes in blood can be detected, offering the opportunity to predict fertility potential through biomarkers (Banerjee et al., 2023a; Marrella and Biase, 2023; Moorey et al., 2020). Moreover, circulating biomarkers may provide insights into the functional status of other tissues, including reproductive organs (Brulport et al., 2024; Fujiwara, 2009). Peripheral blood mononuclear cells have been suggested to positively contribute to the embryo-maternal interaction in early pregnancy by enhancing progesterone production in pregnant women (Fujiwara, 2009). Likewise, the transcriptomic profile of peripheral white blood cells (PWBCs) has been examined in cattle to predict pregnancy outcomes in heifers (Banerjee et al., 2023a; Kertz et al., 2023; Marrella and Biase, 2023). PWBCs are key mediators of the immune response and represent a valuable source of information about maternal immune status (De los Santos et al., 2023). Omics-based, high-throughput technologies, including transcriptomics and proteomics, have been increasingly applied to PWBCs to capture the molecular signatures associated with fertility. Transcriptomic analyses provide insights into gene expression patterns linked to immune regulation, uterine receptivity, and embryo survival, while proteomic profiling offers complementary insights into the functional proteins that mediate these processes (Banerjee et al., 2023a; Marrella and Biase, 2023).

Using multi-omics approaches, Marrella and Biase (2023) identified molecular signatures that discriminate heifers with differing fertility potential at the time of AI. Similarly, we demonstrated that 92 genes were differentially expressed between fertile and subfertile heifers at AI, with immune response and cytokine production pathways over-represented in the subfertile group (Banerjee et al., 2023a). Integrating different omics layers may therefore help uncover the biological causes of subfertility and pinpoint regulatory biomarkers. This approach could ultimately enable earlier and more accurate prediction of pregnancy outcomes in heifers. Based on this, we hypothesized that beef heifers that establish pregnancy following artificial insemination exhibit distinct gene and protein expression dynamics in peripheral white blood cells before AI compared with non-pregnant heifers. Furthermore, we hypothesized that integrative multi-omics and network analyses would reveal a rewiring of key regulatory genes and proteins contributing to divergent reproductive outcomes. Thus, our goal is to identify molecular biomarkers and regulatory networks associated with pregnancy establishment in beef heifers before AI by integrating transcriptomic and proteomic data. By combining transcriptomic and proteomic profiling, along with co-expression network analyses, we uncovered biological pathways and candidate genes and proteins that may contribute to pregnancy success in beef cattle.

## 2 Materials and methods

### 2.1 Ethics approval

All experimental procedures involving animals were conducted with approval from the Institutional Animal Care and Use Committee (IACUC) at Auburn University (protocol number 2021-3968).

### 2.2 Heifer reproductive management, sample collection, and fertility classification

Simmental-Angus cross-bred heifers (n = 92) included in this research were developed for breeding replacements at Auburn University, Alabama Agricultural Experiment Station. To investigate differences in fertility potential and pregnancy outcomes, developing heifers were assessed for reproductive tract score (RTS), weight, and age at artificial insemination (AI). To investigate the pubertal status of each heifer, ∼30 days before the breeding season, the RTS and pelvic measurements were assessed as previously described (Phillips et al., 2018). The RTS is a 1–5 scale used to evaluate pubertal development and reproductive readiness in beef heifers. Animals were classified from immature/prepubertal (RTS 1–2) to cycling/mature (RTS 4–5) according to the criteria described by Gutierrez et al. (2014).

These heifers were exposed to their first breeding season at approximately 437 days of age and subjected to a fixed-time AI protocol (7-Day CO-Synch + CIDR) (Dickinson et al., 2018). Heifers received 100 µg of GnRH (Cystorelin; Merial Inc., Duluth, GA, United States) intramuscularly, and a slow-release device containing 1.38 g of progesterone (Eazi-Breed CIDR; Zoetis Inc., Kalamazoo, MI, United States) was inserted intravaginally. CIDRs were removed 7 days later, and heifers received a prostaglandin F2α intramuscular injection (dinoprost tromethamine 25 mg, Lutylase, Zoetis Inc., Kalamazoo, MI, United States). Artificial insemination was performed in all heifers using one straw of semen from a single Angus sire approximately 54 h after the CIDR removal. They also received a 100 µg injection of GnRH at the time of AI. Fourteen days after AI, heifers were placed with bulls for natural service for three consecutive breeding cycles (approximately 60 days). The bulls had undergone and passed a breeding soundness examination before the breeding season. Pregnancy status was determined via transrectal ultrasonography 30 days following the end of the natural breeding season. Blood samples were collected from heifers 2 days prior to AI from the jugular vein.

Blood was collected (10 mL) into vacutainers containing EDTA (K2EDTA 18 mg, BD Vacutainer®, Franklin Lakes, NJ, United States). These samples were processed shortly after collection, and peripheral white blood cells (PWBCs) were isolated as reported elsewhere (Dickinson et al., 2018) and stored at −80 °C until further analysis.

**FIGURE 1:**
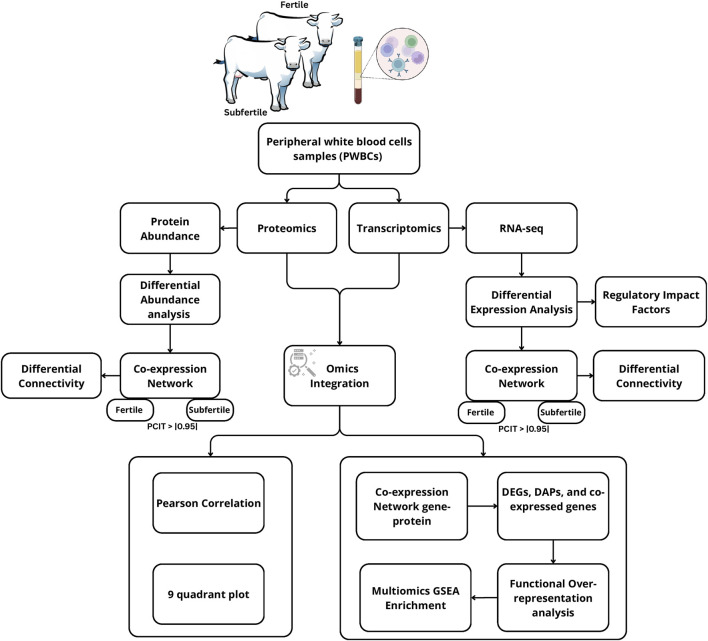
Diagram illustrating the study design and data integration analyses of transcriptomics and proteomics datasets from peripheral white blood cells of subfertile and fertile heifers.

Heifers were retrospectively categorized as either fertile or subfertile after determining the outcomes of AI and natural service. Heifers were selected within the group that conceived from the first AI, while subfertile heifers were defined as those that failed to conceive after AI and an additional 60-day period of natural breeding (non-pregnant group). Subfertile heifers were clinically healthy, cycling, and reproductively sound throughout the breeding program. Within each stratum, heifers were selected considering the phenotypic similarities (RTS ≥4; body weight∼850 lbs, and age at breeding) to minimize confounding effects due to known sources of biological variability. We selected six heifers per group for further molecular analyses. Figure 1 summarizes the experimental design and analysis workflow.

### 2.3 RNA isolation and sequencing

Total RNA isolation from the PWBCs was performed in a single batch following the Trizol reagent (Invitrogen, Carlsbad, CA, United States) protocol, as previously described (Banerjee et al., 2023a). RNA purification was performed using the RNA Clean and Concentrator kit (Zymo Research, Irvine, CA, United States) with DNase I digestion. Quality assessment and RNA integrity were performed using the Qubit RNA BR and RNA IQ Assay Kits on a Qubit Fluorometer v4.0 (Thermo Fisher Scientific Inc., MA, United States). Directional mRNA libraries were prepared from all samples using poly(A) selection and the NEBNext Ultra II Ultra II Directional RNA Library Prep Kit (New England BioLabs, Ipswich, MA, United States), followed by paired-end 150-bp sequencing on an Illumina NovaSeq platform (Novogene Co., Nanjing, China) at a depth of ∼20 million reads per sample. Sequencing was performed in a single run over two lanes with samples evenly distributed according to the fertility classification.

### 2.4 Data quality control, mapping, and differential expression analysis

Raw data quality control was performed using FastQC v0.11.9 (Andrews, 2010), and output files were aggregated with MultiQC v1.12 (Ewels et al., 2016) to determine quality scores, per sequence GC content, and adapter content. Read mapping was performed to the *Bos taurus* reference genome (ARS UCD1.3) (Rosen et al., 2020) using the STAR aligner v2.7.5 (Dobin et al., 2013). Gene counts were determined using the *-quantMode* geneCounts option in STAR, with the *B*. *taurus* annotation file from Ensembl (release 109).

The *filterByExpr* function from edgeR v4.0.14 was used to remove lowly expressed genes (minimum 10 counts per million in 70% of samples) (Robinson et al., 2010). An ANOVA and a principal component analysis (PCA) using the stats4 v3.6.3 and factoextra v1.0.7 R packages (Kassambara and Mundt, 2017), respectively, were implemented to test for potential technical biases. To identify differentially expressed genes between subfertile and fertile groups, we used a negative binomial generalized linear model implemented in DESeq2 v.1.42.0 (Love et al., 2014).

The differential analysis was based on the design model, considering the groups (subfertile vs. fertile) to estimate gene count dispersion and log2 fold changes (log2FC) (Love et al., 2014). The Wald test was used to determine statistical significance, and genes were deemed significant if *P* ≤ 0.05 and the absolute log_2_ fold change (|log2FC|) ≥ 0.5. The direction of regulation (up or downregulation) was assigned considering the sign of the log2FC in the subfertile group. Gene annotation was performed using BiomaRt v2.58.1 (Durinck et al., 2009). The distribution of DEGs was visualized using a volcano plot generated with the EnhancedVolcano v1.4.0 R package (Blighe et al., 2024).

### 2.5 Proteomics data generation and differential abundance analysis

The PWBCs were used for proteome profiling conducted by BGI Genomics (San Jose, CA, United States) in a single batch. To this end, cells were resuspended in lysis buffer (5% sodium dodecyl sulfate, SDS, Sigma-Aldrich, MO, United States; and 9 M urea buffer, Invitrogen, Carlsbad, United States; in 50 mM triethylammonium bicarbonate, TEAB; Thermo Scientific, MA, United States), followed by the bicinchoninic acid (BCA) assay for the detection and quantitation of total protein. Next, samples were processed following the S-Trap™ Universal Sample Prep Mini Kit (ProtiFI, Fairport, NY, United States). To this end, 20 μg of each sample was reduced with dithiothreitol (DTT, 10 mM; Thermo Scientific, MA, United States) and alkylated with iodoacetamide (IAM, 20 mM; Thermo Scientific, MA, United States). The resulting samples were digested overnight using the S-Trap™ Micro Spin column (ProtiFI, Fairport, NY, United States) with Trypsin/Lys-C. Samples were eluted following the S-Trap™ protocol and then dried in speed-vacuum (Speed-Vac). Reconstitution of the samples was performed using mobile phase A (2% acetonitrile, 0.1% formic acid). Each sample (500 ng of peptides) was loaded onto the nano-flow liquid chromatography and high-resolution Orbitrap mass spectrometry (nano LC-MS/MS; Thermo Fisher Scientific) system with a 2-h gradient. The LC was operated at a flow rate of 0.3 μL/min. The Orbitrap Eclipse MS was set to an ion spray voltage of 2100 V, with the FAIMS Pro™ operated using three compensation voltages (−50, −60, and −85 V). To minimize potential run-order and technical bias, samples from fertile and subfertile groups were randomized prior to loading and data acquisition on the mass spectrometer.

Mass spectrometry data and database searching were performed using Proteome Discoverer 2.5 (Thermo Scientific, MA, United States) with SEQUEST HT analysis against the reviewed Bovine UniProt database (May 2023). This algorithm allows for the assignment of the MS/MS spectra to peptide database sequences. High (<1%), medium (<5%), and low (>5%) confidence levels were assigned to protein identifications based on the False Discovery Rate (FDR), which is an estimate of the proportion of incorrect predictions. Protein abundance was determined as the sum of its associated peptide group abundances.

We performed a differential abundance analysis to identify significant changes between groups. To this end, only proteins with high or medium confidence were used (n = 5,094 out of 6,738). Protein abundance data were log2-transformed and normalized using median normalization. Differentially abundant proteins (DAPs) were determined using a moderated t-test based on the limma package (Ritchie et al., 2015) implemented on protti v0.7.0 R-package (Quast et al., 2022) comparing non-subfertile vs. fertile groups (*P* ≤ 0.05). The subfertile group (subfertile/fertile) was used as the reference. Protein annotation was performed using BiomaRt v2.50.3 (Durinck et al., 2009). The distribution of DAPs was visualized through a volcano plot (Blighe et al., 2024).

### 2.6 Co-expression network and data integration analyses

We used co-expression and differential co-expression approaches based on the PCIT (Partial Correlation and Information Theory) and RIF (Regulatory Impact Factors) algorithms to prioritize candidate regulatory genes and proteins (Reverter et al., 2010; Reverter and Chan, 2008). To this end, the analyses were performed considering each omics individually, and then the transcriptome and proteome datasets were integrated. Separate networks were constructed for fertile and subfertile heifers. Normalized gene expression obtained using the VST function in DESeq2 (Love et al., 2014), and median-normalized (Quast et al., 2022) protein abundance were used as inputs for network inference. Proteins with missing values were filtered out. Significant co-expression pairs were prioritized using a correlation threshold r ≥ |0.95| for both proteins and genes. In addition, gene pairs were selected when they contained at least one DEG or RIF. Gene and protein networks were visualized in Cytoscape (Shannon et al., 2003). The changes in the nodes and edge rewiring between groups were visualized using DyNet Cytoscape plug in (Goenawan et al., 2016).

For the RIF approach, only the normalized abundance of DEGs, as described above, was used. This analysis identifies regulators (transcription factors, TFs) whose connectivity changes between two groups, in this case, subfertile vs. fertile groups, even though they may not be differentially expressed (Pérez-Montarelo et al., 2012). The impact factor was estimated considering the RIF1 and RIF2 metrics. RIF1 emphasizes TFs showing significant differential co-expression and expression differences between groups, while RIF2 highlights TFs whose expression better predicts DEG abundance (Reverter et al., 2010).

We mined the Animal Transcription Factor Database (Animal TFDB v4.0) (Shen et al., 2023) from which we downloaded 1,445 TFs annotated from *B. taurus*. Furthermore, these TFs were then cross-referenced with the list of expressed genes, and only those expressed were kept for further analysis. We first performed RIF analysis using TF gene expression as modulators and DEGs as targets to predict gene-gene regulation. Next, we investigated whether these regulatory TFs were among the expressed proteins or the DAPs list. Potential regulators (RIF1 or RIF2) were deemed significant considering a RIF score greater than |1.96| of the standard deviation (SD, *P* ≤ 0.05) (Reverter et al., 2010).

We implemented a differential connectivity analysis to investigate gene or protein interaction rewiring between the two groups. To this end, networks were predicted using PCIT as described above. To measure the differential connectivity (*DK*), we used the method proposed by Fuller et al. (2007): ${DK}_{i}=K_{fertile\;\left(i\right)}-K_{subfertile\;\left(i\right)}$ , where: $k_{fertile\;\left(i\right)}$ and $k_{subfertile\;\left(i\right)}$ represents the normalized connectivity of the *ith* gene in the whole network of fertile and subfertile groups, respectively. Significance was determined based on *z-scores*. Values greater than ±1.96 SD were deemed significant (*P* ≤ 0.05), as previously described (Diniz et al., 2022).

Lastly, we examined patterns by integrating the transcriptome and proteome datasets. To this end, we employed a nine-quadrant plot to visualize the distribution of fold changes in gene and protein abundance between fertile and subfertile heifers. To determine the concordance between transcriptomic and proteomic changes, we calculated the Pearson correlation of fold changes using only the features present in both datasets. Furthermore, to retrieve meaningful gene-protein interactions, we used the PCIT algorithm to measure correlation between co-expressed pairs. Pairs in which at least one member was a DEG or DAP were retained for further analysis (|r| ≥ 0.90|, *P* ≤ 0.05).

### 2.7 Gene and protein functional over-representation analysis

To characterize the biological functions of DEGs, DAPs, and co-expressed genes, an over-representation approach was used to identify KEGG pathways and biological processes (BPs). To this end, these lists were used individually for over-representation analyses implemented on the WebGestalt 2024 web tool (Elizarraras et al., 2024). Significantly over-represented terms were identified at *P* ≤ 0.05, considering all expressed genes and all proteins after quality control and with Ensembl IDs mapped to *B*. *taurus*. For the multi-list analysis, the genome protein-code from *B*. *taurus* was used as background.

### 2.8 Multi-omics gene set enrichment analysis

To assess the relationships across all expressed genes (n = 13,012) and proteins (n = 5,094) remaining after quality control to a collection of pre-defined sets underlying the KEGG and REACTOME pathways, we carried out a multi-omics gene set enrichment analysis (multiGSEA). This approach was implemented using the multiGSEA v1.18.0 R package (Canzler and Hackermüller, 2020), which applies the GSEA algorithm from the fGSEA package individually for each omics layer and then derives a composite multi-omics pathway analysis (Korotkevich et al., 2016). To provide a comprehensive overview, multiGSEA computes and aggregates p-values across multiple omics layers. To this end, a normalized enrichment score (NES) was calculated based on a pre-ranked list of features that accounts for the direction of the fold change and the magnitude of its significance. The ranking was defined by the equation: $rank=[sign\;\left(\log2FC\right)\times -\log\;10(p-value$ )]. The NES represents the strength and direction of association between a gene set and over-represented pathways. Pathways with a positive NES are enriched for upregulated (top-ranked) genes, whereas pathways with a negative NES are enriched for downregulated genes in the subfertile group. Then, multiGSEA computed and combined p-values using Stouffer’s method, which is not biased towards small or large p-values (Canzler and Hackermüller, 2020). Pathways were considered significant when *P* ≤ 0.01 and |NES| ≥ 1.5.

## 3 Results

We implemented a multi-tiered approach combining multi-omics and network analyses to reveal the rewiring of key regulatory genes and proteins underlying divergent reproductive outcomes in beef heifers. Additionally, we investigated biological pathways, candidate genes, and proteins that contribute to pregnancy success in beef cattle.

### 3.1 Transcriptomics and proteomics analyses reveal differences between subfertile and fertile heifers

We generated transcriptomic and proteomic profiles from PWBCs of 12 beef heifers 2 days before AI, retrospectively classified as subfertile or fertile based on pregnancy outcomes. The overall pregnancy rate for the herd was 83.7%. Heifers classified as fertile had an average age at AI of 14.51 ± 0.54 months, an RTS of 4.16 ± 0.40, and a body weight of 845.66 ± 30.97 lbs. Similarly, subfertile heifers had an average age at AI of 14.55 ± 0.37 months, an RTS of 4.16 ± 0.40, and a body weight of 851.33 ± 50.95 lbs.

**FIGURE 2:**
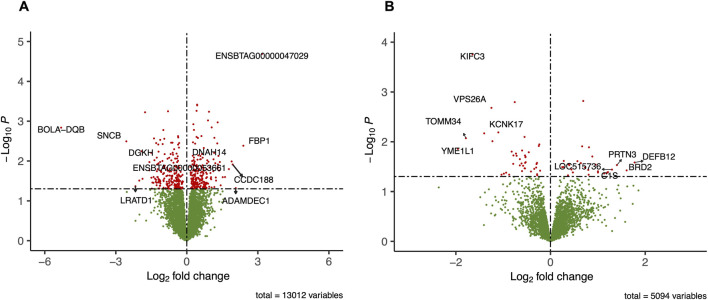
Volcano plot of differentially expressed genes **(A)** and differentially abundant proteins (DAPs) **(B)** from peripheral white blood cells of subfertile and fertile heifers. Dots indicate individual genes or proteins. The x-axis shows the log2 fold change between the fertile and subfertile groups. The -log (base 10) of the *P* is shown on the y-axis. Color coding indicates significance: red for DEGs meeting P ≤ 0.05 and |log2 fold change| ≥ 0.5 or proteins (*P* ≤ 0.05), and blue, green, or grey for non-significant genes. Up and downregulation of genes/proteins was assigned based on the sign of the log2 fold change in subfertile heifers. The horizontal dashed line indicates the significance threshold (*P* ≤ 0.05), and the vertical dashed lines represent the fold change cutoff for up and downregulated genes (|log2FC| ≥ 0.5).

The RNA-Seq generated on average 27.87 million reads per sample, ranging from 22 to 33 million clean reads. A summary of the read statistics and mapping rates was provided in the Supplementary Table S1. The mapping of cleaned reads to the *B. taurus* ARS-UCD 1.3 genome assembly resulted in an average 94.2% (26.26 M) of mapped reads. After removing lowly or non-expressed genes using edgeR, we kept 13,012 genes out of 27,607 for further analysis. Through a differential expression analysis using a negative binomial model implemented in DESeq2, we identified 230 DEGs, of which 128 and 102 were downregulated and upregulated in the subfertile group, respectively (Figure 2A; Supplementary Table S2). Among the DEGs, 13 were TFs, including *L3MBTL1, MEIS1, PAX5*, and *TSC22D3*. The functional classification of DEGs was as follows: 216 protein-coding genes, 6 long non-coding RNAs, 3 pseudogenes, 4 small nucleolar RNAs, and 1 microRNA. The top five upregulated genes, based on log2FC, were *ENSBTAG00000047029, FBP1, ADAMDEC1, CCDC188,* and *DNAH14*. Likewise, the top five downregulated genes were *BOLA-DQB, SNCB, LRATD1, ENSBTAG00000053661,* and *DGKH.*

Using untargeted LC-MS/MS proteomic profiling, we identified 6,738 proteins. After filtering out proteins assigned to a low confidence score, 5,094 remained, including 166 TFs (Supplementary Table S3). The differential abundance analysis between subfertile and fertile groups resulted in 70 DAPs (*P* ≤ 0.05), of which 43 were downregulated, and 27 were upregulated in the subfertile group (Figure 2B; Supplementary Table S3). The top five proteins showing the largest fold-change differences between groups were DEFB12, BRD2, PRTN3, LOC515736, and C1S (upregulated), and YME1L1, TOMM34, KIFC3, KCNK17, and VPS26A (downregulated). Additionally, differential abundance analysis revealed that BACH1 was not only significantly downregulated in the subfertile group but also function as a transcription factor.

**FIGURE 3:**
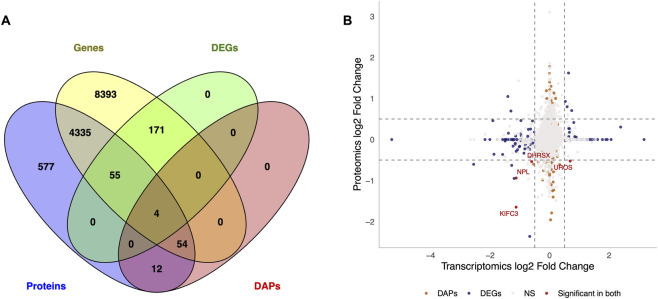
Integration of transcriptomic and proteomic datasets from peripheral white blood cells of subfertile and fertile heifers. **(A)** Venn diagram representing the overlap between genes and proteins identified across datasets, including those differentially expressed (DEGs) or differentially abundant (DAPs); **(B)** Nine-quadrant plot showing the relationship between transcriptome and proteome. The x-axis and y-axis represent transcriptomic and proteomic log2 fold changes (subfertile vs. fertile). Each point corresponds to a gene-protein pair matched by Ensembl ID. Quadrants illustrate the direction and magnitude of change at both the transcript and protein levels. The upper-right and lower-left quadrants indicate molecules that change in the same direction in both datasets. The upper left and lower right quadrants represent opposite regulatory patterns between transcript and protein levels. Dashed lines mark the log2 fold change thresholds (±0.5). Red dots represent molecules significant in both datasets (shared DEGs and DAPs), dark navy blue indicates significance only at the transcript level (DEGs; *P* ≤ 0.05 and absolute (log2 fold change ≥0.5)), and dark orange indicates significance only at the protein level (DAPs; *P* ≤ 0.05), grey dots represent genes or proteins not differentially expressed. The Venn diagram was created using Venny v.2.1 (Oliveros, 2000).

Interestingly, the intersection between the transcripts (13,012) and proteins (5,037 unique IDs), based on the Ensembl IDs, identified 4,502 features (∼33%; corresponding to 4,448 unique IDs) that intersected between datasets (Figure 3A). For the overlapping transcripts and proteins, a substantial fraction of features exhibited concordant changes (up-up: 1,291; down-down: 1,195), whereas 2,016 features showed discordant changes (up-down: 901; down-up: 1,115) according to the sign of the log2FC (Figure 3B). The correlation between genes and proteins’ log2FC was low (r = 0.05; *P* = 3.74e-08). Despite some differences, most transcript and protein abundance changes were consistent due to fertility status. Except for *UROS*, which was upregulated at the transcript level but downregulated at the protein level, *KIFC3, DHRSX,* and *NPL* were consistently downregulated in subfertile heifers across both datasets.

### 3.2 Co-expression networks identify regulatory genes and proteins

The potential regulatory effect of each transcription factor on the DEGs was estimated using the differential co-expression concept and the RIF metrics (RIF1 and RIF2). We identified 926 TFs expressed among the 13,012 genes. These TFs were then contrasted against the 230 DEGs. We identified 92 TFs that may play a regulatory role in gene expression differences (*P* ≤ 0.05; Supplementary Table S4). However, among them, only 18 were observed on the proteomics dataset. Interestingly, TFs previously related to immunity were among the potential regulators, including *IRF7*. In addition, TFs associated with epigenetic regulation and chromatin remodeling (*BACH1*, *L3MBTL1, MDB1, MDB2,* and *SMARCE1)* were observed at the gene and protein levels.

**FIGURE 4:**
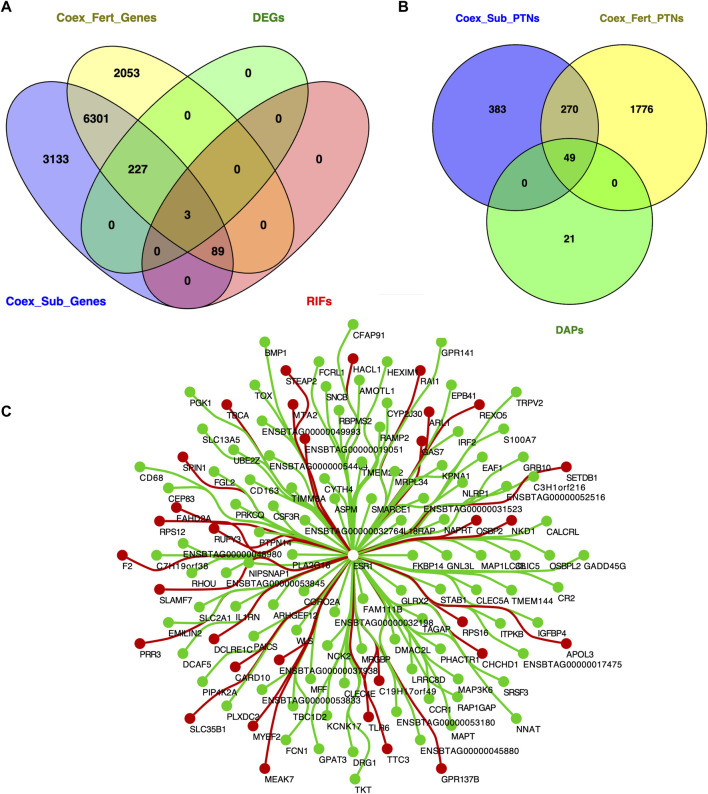
Overlapping genes and proteins among analyses from peripheral white blood cells of subfertile and fertile heifers. **(A)** Overlapping differentially expressed genes (DEGs), differentially connected genes (DK), and gene regulators (RIFs) with expressed proteins (PTNs); **(B)** Overlapping between Differentially abundant proteins (DAPs) and co-expressed proteins between fertile and subfertile groups. The Venn diagram was created using Venny v.2.1 (Oliveros, 2000); **(C)** Central reference union networks between the fertile and subfertile groups, with 2,478 nodes (proteins) and 4,165 edges (interactions). Unique nodes in fertile and subfertile heifers are shown in green and red, respectively. White nodes are shared between groups. The central reference network was constructed using DyNet (Goenawan et al., 2016).

Next, we investigated the co-expression patterns within groups and across omics datasets to explore relationships among molecules and to determine changes in network topology associated with fertility status. The co-expression analysis of 13,012 genes yielded 1,839,518 significant connections in the fertile group and 2,087,171 connections in the subfertile group (*P* ≤ 0.05). To identify biologically relevant patterns and reduce the complexity of the data, we selected genes showing absolute correlation coefficients greater than 0.95 and co-expression with DEGs or RIFs (*P* ≤ 0.05). After filtering, 23,374 (corresponding to 8,673 unique genes) and 27,662 (corresponding to 9,753 unique genes) significantly co-expressed gene pairs were identified in the fertile and subfertile groups, respectively, with 6,620 genes common to both groups (Figure 4A; Supplementary Table S5; Supplementary Figure 1). The 92 RIFs were shared between both co-expression networks, including *MAFF (z-score = 3.10), MEIS1 (z-score = 3.3),* and *L3MBTL1 (z-score = 2.59)*. We also ranked the genes to identify differences in their connectivity measures (*DK*) between groups. We identified 203 differentially connected genes (*P* ≤ 0.05), with 147 genes showing reduced connectivity in the subfertile group, of which 69 were TFs (Supplementary Table S6). By overlapping the lists, we found that *MAFF* and *MEIS1* TFs were downregulated in the subfertile group and had significantly fewer connections than in the fertile group. The TF coding gene *ESR1* was identified as a potential regulator based on RIF1 and RIF2 metrics. Additionally, *ESR1* was significantly more connected in the fertile group (99 vs. 32 connections; Figure 4C; Supplementary Table S5). Positive correlation between *ESR1* and *IRF2, SMARCE1* and *NNAT* were observed in the fertile group. On the other hand, negative correlations included *CSF3R, SRSF3*, and *BMP1*.

Regarding the protein-protein co-expression networks, we used 4,627 proteins to create networks separately for each group. Based on PCIT, we identified 712,571 significant connections in the fertile group and 435,400 connections in the subfertile group (*P* ≤ 0.05). After filtering (|r = 0.95| and co-expressed with DAPs), 3,448 (corresponding to 2,095 unique proteins) and 722 (corresponding to 702 unique proteins) correlated protein pairs were identified in the fertile and subfertile groups, respectively, with 319 proteins common to both groups (Figure 4B; Supplementary Table S7; Supplementary Figure 2). The connectivity analysis yielded 34 *DK* proteins (*P* ≤ 0.05), with 18 more connected in the subfertile group and 16 less connected (Supplementary Table S8). Among the co-expressed and *DK* overlapping proteins between groups were BACH1, UROS, DHRSX, and NPL.

Lastly, we determined the co-expression patterns between genes (n = 13,012) and proteins (n = 4,627) within fertility groups. We identified 2,963,829 and 3,015,844 significant pairs from the fertile and subfertile network groups, respectively. Only co-expressed pairs in which at least one member was a DEG or DAP were retained for further analysis (|r| ≥ 0.90|, *P* ≤ 0.05), resulting in 13,799 and 18,083 pairs in fertile and subfertile groups. Interestingly, most gene pairs were group-specific, with only 149 shared between groups. A total of 17,934 pairs were unique to the subfertile group (5,101 genes and 4,048 proteins), and 13,650 (4,297 genes and 3,308 proteins) were unique to the fertile group (Supplementary Table S9). For the shared pairs, we examined the direction of correlation between groups and found that 83 pairs exhibited a change in correlation (from positive to negative or *vice versa*).

### 3.3 KEGG pathways and biological processes are differentially modulated between fertility groups

**FIGURE 5:**
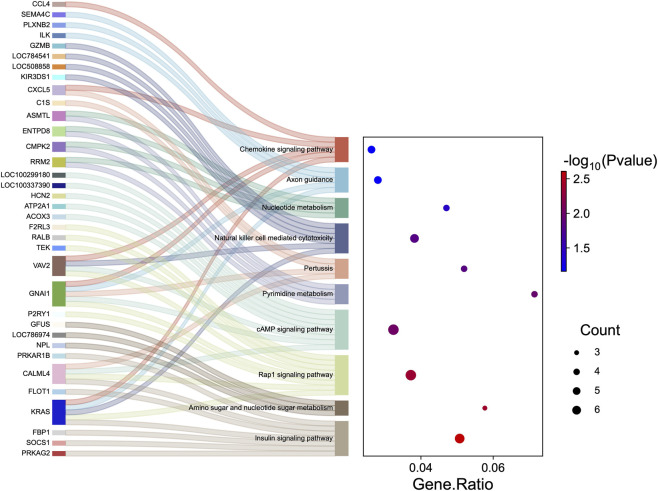
Sankey and dot plots illustrate overlapping pathways associated with differentially expressed genes (DEGs) and differentially abundant proteins (DAPs) from peripheral white blood cells of subfertile and fertile heifers. The Sankey plot shows the connections from individual molecules to their respective pathways, while the dot plot quantifies the pathway enrichment. Colors in the Sankey plot were randomly assigned. In the dot plot, the color scale represents −log10 (p-value), and the dot size indicates the number of over-represented molecules in each pathway.

To evaluate functional differences between groups, we performed over-representation and GSEA analyses across multi-omics datasets. The pathways and biological process (BP) terms over-represented from the DEG list yielded ten GO terms shared between DEGs and DAPs (meta-p ≤ 0.01). Among these, terms such as chromosome organization, regulation of the cell cycle, and protein-DNA complex organization were identified through meta-analysis by aggregating both DEG and DAP lists (Supplementary Table S10). At the protein level, BPs observed exclusively included positive regulation of interferon-beta (IFNβ) production in addition to those related to plasma membrane organization (*P* ≤ 0.01). Similarly, the meta-pathway analysis included insulin signaling, Rap1 signaling, and chemokine signaling pathways (Figure 5; Supplementary Table S11).

**FIGURE 6:**
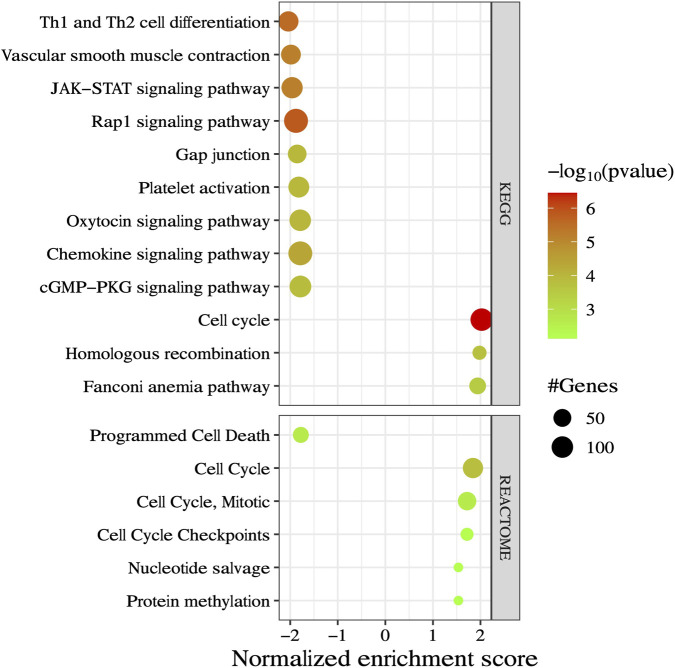
Pathway over-representation analysis based on gene set enrichment of expressed genes and proteins from peripheral white blood cells of fertile and subfertile heifers. The normalized enrichment score (NES) of top-ranked (positive NES) and bottom-ranked (negative NES) pathways based on the contrast between subfertile vs. fertile heifers. Significant pathways were defined by thresholds of *P* ≤ 0.01 and NES ≥ |1.5|.

A gene set enrichment analysis was applied to rank all expressed genes and proteins, and identify associated biological pathways based on a combination of *P* and log2FC. The normalized enrichment score was used to identify significant pathways in the KEGG and REACTOME databases (*P* ≤ 0.01 and NES ≥ |1.5|). We identified 73 and 10 significant pathways based on the gene and protein lists (Supplementary Table S12). No significant meta-pathway was identified by integrating the lists (*P* ≥ 0.01). Pathways such as the Fanconi anemia pathway, homologous recombination, and cell cycle were among the top enriched (positive NES), whereas Th1 and Th2 cell differentiation (*BOLA*-DQB), JAK-STAT signaling pathway (*SOCS1*), and Rap1 signaling pathway (*P2RY1, RALB, F2RL3,* and *CALML4*) were ranked among the top depleted (negative NES) ones in the subfertile. Figure 6 shows the top pathways with the greatest NES values.

## 4 Discussion

Fertility is a multifactorial trait and a key determinant of the sustainability and profitability of the beef industry (Kertz et al., 2023). By integrating transcriptomic and proteomic profiling and co-expression networks from PWBCs, we identified biological pathways, candidate genes, and proteins that contribute to pregnancy success in beef heifers. We identified molecular differences in blood, which offer an opportunity to predict fertility potential through biomarkers. Moreover, PWBCs have been suggested to positively contribute to the embryo-maternal interaction in early pregnancy by enhancing progesterone production in pregnant women (Fujiwara, 2009). Likewise, PWBCs are key mediators of the immune response and represent a valuable source of information on maternal immune status (De los Santos et al., 2023). Herein, we characterized the molecular profiles of beef heifers 2 days before AI and identified 230 DEGs and 70 DAPs between subfertile and fertile groups. These molecules were associated with key pathways and biological processes, including the cell cycle, metabolism (insulin signaling and nucleotide metabolism), and immune-related pathways (chemokine signaling and JAK-STAT signaling). Furthermore, we identified 92 RIF genes with potential regulatory roles in modulating transcriptomic differences between fertile and subfertile heifers. Other studies have investigated the transcriptomic profile of PWBCs in cattle as a predictor of pregnancy outcomes in heifers and have reported several DEGs (Banerjee et al., 2023a; Kertz et al., 2023; Marrella and Biase, 2023). However, proteomics studies are less common.

The number of genes detected by RNA-Seq was about 2.5 times greater than the number of proteins identified by mass spectrometry. Interestingly, we observed limited overlap between the total transcripts and proteins (4,448), as well as common to DEGs and DAPs (*UROS*, *KIFC3, DHRSX,* and *NPL*). From a differential expression perspective, this suggests that post-transcriptional mechanisms play a significant role and warrant further investigation. Based on a similar experimental design, FTO protein and *APMAP* and *DNAI7* transcripts were the only differentially abundant molecules between fertile and subfertile heifers (Marrella and Biase, 2023). In our study, among overlapping transcripts and proteins, a substantial fraction showed concordant changes between subfertile and fertile heifers, as indicated by the sign of log2FC. Similarly, the weak correlation between gene and protein abundance levels based on the log2FC is consistent with previous reports, highlighting the challenges and inherent differences between the two approaches (Backman et al., 2019; Liu et al., 2016). Some of these discrepancies are related to the sensitivity of mass spectrometry in detecting low-abundance molecules (Wang et al., 2019) and the presence of multiple isoforms originating from the same gene through alternative splicing (Liu et al., 2016).

Network analysis revealed distinct regulatory patterns between fertile and subfertile heifers at both the transcriptome and proteome levels. At the gene level, the subfertile group has shown a greater number of connections and unique correlated genes compared to the fertile group, suggesting enhanced transcript-level connectivity. This increased transcriptional co-regulation may reflect a compensatory molecular response or rewiring of the gene network to maintain coordinated gene expression (Banerjee et al., 2023a; van Dam et al., 2017). These results align with our observation that 69 out of 92 RIF genes were among the DK, and 43 of them lost connections in the subfertile group. A similar trend was observed for DEGs that were also identified among the DK genes. In contrast, the proteomic co-expression networks demonstrated a different pattern. The network from fertile heifers showed higher network density and connectivity, suggesting greater functional coordination among proteins. The reduction in co-expressed pairs and connections in the subfertile group suggests post-transcriptional or translational regulation mechanisms (Vogel and Marcotte, 2012). The overlap between group networks highlights the complex multi-layered control of fertility-related pathways, where transcriptional signals are often modulated by subsequent regulatory processes (Liu et al., 2016). Likewise, this rewiring involves changes in their interactions, creating new co-expression patterns that may be linked to an adaptive response (Sharma et al., 2021), as we observed changes in correlation between groups (from positive to negative or *vice versa*). These findings reinforce the concept that transcript abundance alone only partially accounts for protein abundance (Liu et al., 2016; Vogel and Marcotte, 2012), emphasizing the importance of adopting an integrated multi-omics approach as applied in this study.

Among the rewired TFs, we identified *ESR1* at the gene level, while others involved in epigenetic regulation and chromatin remodeling (*BACH1*, *L3MBTL1, MDB1, MDB2, SRF,* and *SMARCE1*) were observed at the gene and protein levels. Estrogen signaling plays a central role in regulating reproductive function, influencing ovarian folliculogenesis, uterine receptivity, and embryo development (Kowalski et al., 2004). Likewise, progesterone-mediated oocyte maturation pathway was over-represented among the differentially abundant proteins, including KRAS and GNAI1. ESR1 acts as a transcriptional regulator mediating these effects by binding to estrogen response elements and modulating the expression of target genes (Hewitt and Korach, 2002). ESR1 plays a key role in regulating luteolysis and the return to cyclicity in non-pregnant cattle. During early pregnancy, IFN-τ suppresses ESR1 expression, thereby inhibiting the secretion of PGF_2_α and extending the lifespan of the corpus luteum, thereby maintaining luteal function (Bazer, 2013). Additionally, ESR1 genetic variants were associated with pregnancy loss in women (Bahia et al., 2020). However, we should consider the physiological differences between humans and cattle when interpreting the role and function of estrogen across species. Although *ESR1* was not among our DEGs or DAPs, it was predicted as a potential regulator of differential expression (RIF), and we found it less connected (*DK*) in the subfertile group (99 vs. 32 connections). Among the co-expressed pairs, *NNAT* was positively correlated with *ESR1* and upregulated in the fertile group. *NNAT* has been reported to be an imprinted gene involved in glucose transport through activation of the PI3K-Akt2 signaling pathway (Kim et al., 2007). This pathway has also been proposed to function as a non-gonadotropic regulator of follicle growth and survival, with evidence that deletion of several of its component genes is associated with infertility (Dupont and Scaramuzzi, 2016; Gareis et al., 2020). Banerjee et al. (2023a) identified *ESR1* as downregulated and an exclusive hub gene in the PWBCs of subfertile heifers at weaning. Additionally, they reported that it was modulated by bta-miR-1839 (Banerjee et al., 2023b). Thus, the rewiring of this gene in the subfertile group may contribute to the regulation of key genes involved in pregnancy recognition failure. Beyond its direct transcriptional role, ESR1 interacts with multiple epigenetic and chromatin remodeling factors in response to estrogen and growth factors (Magnani and Lupien, 2014). Epigenetic TFs identified included *MBD1* and *MBD2*, which are involved with transcript regulation through DNA methylation and modulating the H3K9me3 histone mark (Li et al., 2015). This interplay highlights a multi-layered regulatory mechanism in which estrogen signaling integrates with the epigenetic machinery to fine-tune gene networks associated with reproductive success. Rewiring of these pathways, as observed in subfertile animals, may therefore reflect compromised coordination between hormone-dependent transcription and chromatin dynamics, ultimately influencing fertility outcomes.

We identified four genes that were shared between the transcriptomic and proteomic expression patterns of fertile and subfertile heifers. *KIFC3, DHRSX,* and *NPL* exhibited concordant regulation between the DEGs and DAPs in both groups. Interestingly, transcriptomic analysis indicated *UROS* upregulation, while proteomic data revealed downregulation, suggesting post-transcriptional regulation or altered protein turnover. Among these genes, Xiao et al. (2013) reported that *NPL* expression was significantly upregulated by progesterone and downregulated by estradiol in the mouse uterine luminal epithelium during the preimplantation period. The same authors suggested that *NPL* is involved in embryo implantation due to its role in the breakdown of sialic acid, which is involved in cell adhesion (Xiao et al., 2013). While *KIFC3* has been suggested to be essential for cytokinesis during oocyte meiosis, *DHRSX* and *UROS* have not previously been associated with fertility, warranting further investigation.

The functional over-representation analyses revealed distinct molecular processes between fertile and subfertile heifers at the transcript and protein levels. Biological processes uniquely enriched in the subfertile group included positive regulation of IFNβ production, suggesting altered immune signaling. Successful pregnancy relies on finely tuned immune adaptations that promote maternal tolerance while maintaining defense against pathogens (Aghaeepour et al., 2017). We previously reported that immune-related processes were dysregulated in subfertile heifers, as indicated by transcriptomic profiles from peripheral white blood cells, endometrial epithelial cells, and caruncular endometrial cells (Banerjee et al., 2023b; 2023a; Diniz et al., 2024; Kertz et al., 2025). Interferons (IFNs) are cytokines that play essential roles in regulating cell proliferation and modulating immune responses (Platanias, 2005). Previous studies have shown an increased risk of fetal loss and low birth weight in women who received IFNβ therapy during the first trimester of pregnancy (Boskovic et al., 2005). Due to their complex regulatory nature, IFNs induce their effects through signaling cascades, including the JAK (Janus-activated kinase)-STAT (signal transducer and activator of transcription) pathway (Platanias, 2005). Additionally, genes associated with the JAK-STAT signaling pathway were over-represented among negatively ranked genes in our GSEA analysis, and *SOCS1*, a key inhibitor of cytokine signaling (Sandra et al., 2005), was downregulated in subfertile heifers. In ewes, Sandra et al. (2005) reported that *SOCS1* is involved in regulating endometrial receptivity and blastocyst attachment. Furthermore, among co-expressed genes, *ERS1* was positively correlated with *IRF2*, but negatively correlated with *CSF3R, CD163, CR2,* and *SRSF3* genes. These findings suggest that estrogen signaling is associated with activation of interferon-regulated pathways (Choi et al., 2001) while simultaneously modulating inflammatory, complement, and leukocyte-mediated responses (Fair, 2015).

Meta-pathway analyses of DEGs and DAPs further highlighted the involvement of Rap1 and chemokine signaling pathways, which were also related to reproduction (Kusama et al., 2014; Sigdel et al., 2021). The Rap1 signaling pathway is involved in essential cellular functions, such as uterine decidualization in rats (Kusama et al., 2014), and is critical for maintaining vascular stability during mouse embryonic development (Chrzanowska-Wodnicka et al., 2015). Chemokines play a key role in modulating immune responses, and altered chemokine signaling has been linked to reduced fertility in cattle, affecting uterine receptivity and early embryonic development (Sakumoto, 2024). We observed that *CCL4* and *CXCL5* genes were downregulated in subfertile heifers. In women, downregulation of *CXCL5* in villous tissue has been associated with recurrent spontaneous abortion (Zhang et al., 2021), whereas in pigs, increased *CCL4* expression in endometrial epithelial cells may promote conceptus-endometrial interactions during early pregnancy (Lim et al., 2018). Still related to immune regulation, we identified Th1 and Th2 cell differentiation as over-represented among negatively ranked genes in subfertile heifers, with *BOLA-DQB* showing the largest downregulation (log2FC) in this group. Th1 cells primarily mediate pro-inflammatory, cell-mediated immunity, whereas Th2 cells support anti-inflammatory, humoral responses (Maeda et al., 2013). Proper Th1/Th2 balance is critical for maternal tolerance of the semi-allogeneic fetus (Wang et al., 2020). In cattle, *BOLA*-DQB, a major histocompatibility complex class II gene, plays a key role in antigen presentation and Th1/Th2 differentiation (Norimine and Brown, 2005). Notably, *BOLA*-DQB was downregulated in PWBCs from beef heifers that became pregnant through natural breeding after failing to conceive via AI (Dickinson et al., 2018), further supporting its potential role in fertility. Collectively, our findings support the hypothesis that altered expression might contribute to the potential immune dysregulation observed in subfertile heifers.

While this study offers novel insights into the transcriptomic and proteomic profiles associated with subfertility in heifers, some limitations warrant consideration. First, the relatively small sample size may have reduced the statistical power to detect subtle differences; however, the experimental groups were well balanced to minimize potential confounding effects. Thus, the findings should be interpreted with caution, and validation in a larger, independent cohort is required to confirm our findings. Second, transcriptomic and proteomic analyses capture different layers of molecular regulation, and the weak correlation observed between transcripts and proteins highlights the influence of post-transcriptional and post-translational modifications, which were not fully explored in this study, but warrants further investigation. Third, transcriptomic measurements were limited to PWBCs. Other reproductive tissues and cell types that could influence fertility, such as luteal tissue or immune cell subpopulations, were not assessed. Future research should include a larger cohort to enhance statistical power and consider longitudinal sampling to capture dynamic changes during the breeding period and early pregnancy. Functional experiments could validate the roles of key immune- and fertility-related genes. Integration of additional omics layers, such as epigenomics and metabolomics, may help elucidate regulatory mechanisms linking transcript and protein abundance. Finally, combining PWBC profiling with targeted analysis of tissue-specific immune cell populations may offer a better understanding of the immune mechanisms influencing fertility in cattle. Ultimately, these insights may pave the way for further research investigating predictive biomarkers and to inform selection approaches to optimize fertility and promote sustainable beef cattle production systems.

## 5 Conclusion

This study offers new insights into the molecular basis of fertility in beef heifers by integrating transcriptomic and proteomic profiles from PWBCs collected before AI. We identified distinct gene expression and protein abundance patterns between fertile and subfertile heifers, highlighting biological pathways associated with immune regulation, metabolism, and cell cycle control. Co-expression network analyses revealed marked differences in connectivity between groups, suggesting a rewiring of key regulators. Several transcription factors and epigenetic regulators, including *ESR1*, *MBD1*, and *MBD2*, were identified as key nodes within these networks, suggesting the involvement of epigenetic mechanisms underlying reproductive success. Collectively, our findings demonstrate that PWBCs are a valuable, non-invasive biological source for assessing systemic molecular changes associated with fertility. By integrating transcriptomic and proteomic data, this study enhances our understanding of the intricate regulatory networks governing reproductive outcomes in beef heifers.

## Data Availability

All relevant data are within the paper and its Supplementary Information files. All RNA-sequencing data is publicly available on NCBI’s Gene Expression Omnibus through GEO Series accession number GSE325134.

## Supplementary material

SUPPLEMENTARY DATA SHEET 1 Significantly co-expressed gene pairs between fertile and subfertile heifer groups.

SUPPLEMENTARY DATA SHEET 2 Significantly co-expressed protein pairs between fertile and subfertile heifer groups.
