# Supplementary figures and images for: Network-based multi-omics approaches to identify molecular signatures associated with pregnancy status in beef heifers
Source: Front Genet. 2026 Apr 20;17:1794156. doi: 10.3389/fgene.2026.1794156 (PMC13135839; doi:10.3389/fgene.2026.1794156)

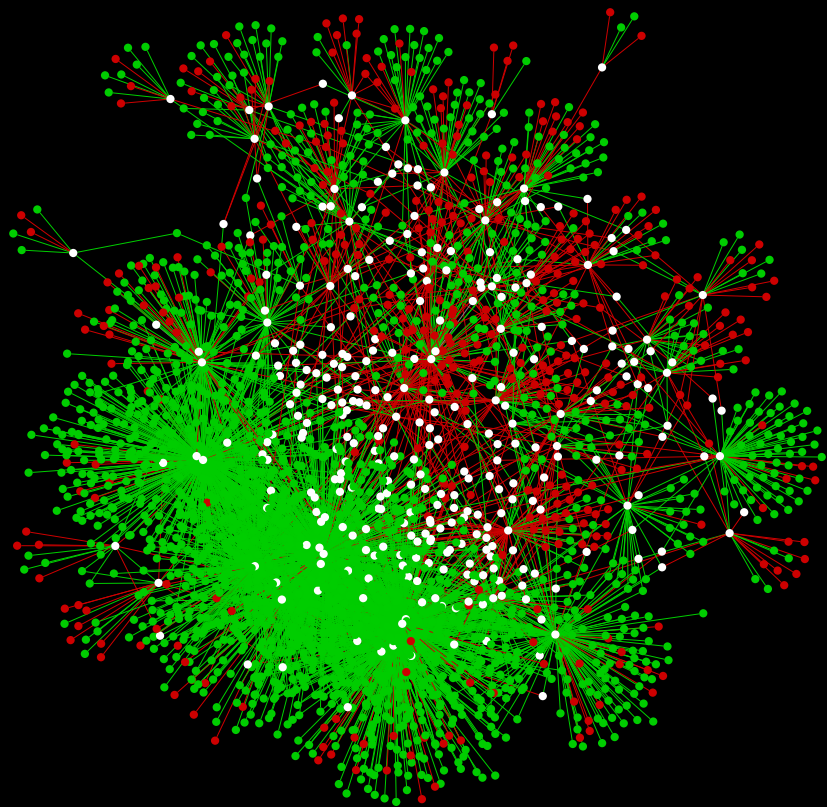

Supplement: Supplementary file 1 [file DataSheet2.pdf]

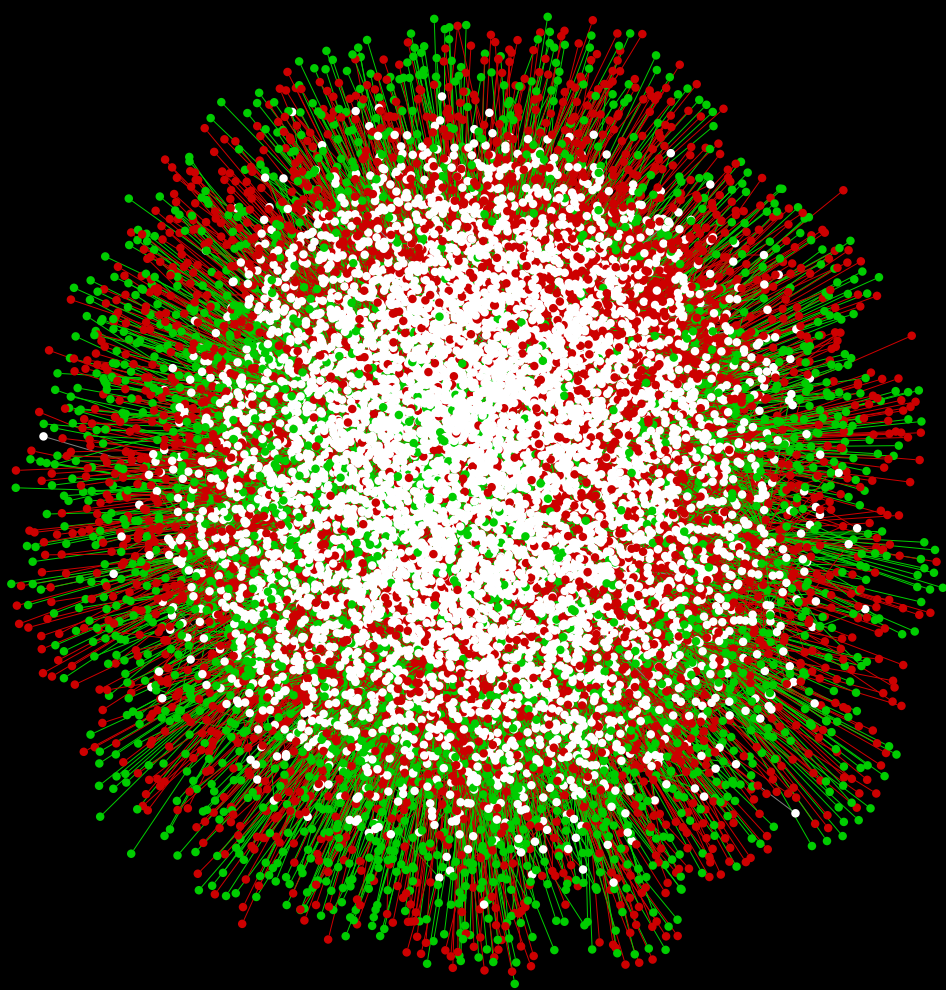

Supplement: Supplementary file 6 [file DataSheet1.pdf]
